# Editorial: Recent advances on renoprotection and kidney regeneration

**DOI:** 10.3389/fphys.2023.1204789

**Published:** 2023-04-27

**Authors:** Ignacio Gimenez, Christian Hugo, Vladimir T. Todorov

**Affiliations:** ^1^ Renal and Cardiovascular Physiopathology (FISIOPREN), Aragon’s Health Sciences Institute, Zaragoza, Spain; ^2^ Institute for Health Research Aragon (IIS Aragon), Zaragoza, Spain; ^3^ School of Medicine, University of Zaragoza, Zaragoza, Spain; ^4^ Experimental Nephrology, Division of Nephrology, Department of Internal Medicine III, University Hospital and Medical Faculty Carl Gustav Carus, Technische Universität Dresden, Dresden, Germany; ^5^ Institute of Physiology, Medical Faculty Carl Gustav Carus, Technische Universität Dresden, Dresden, Germany

**Keywords:** kidney damage, renal regeneration, kidney imaging, diagnostic markers, organ perfusion, ex vivo models

Although several modes of renal replacement therapy have been co-existing for decades, end-stage renal disease (ESRD) represents a huge global healthcare burden. ESRD prevalence increased by over 40% between 2003 and 2016 (Thurlow et al., 2021). Pandemic diseases such as hypertension, obesity, and diabetes where ESRD is a frequent co-morbidity essentially contribute to this increase. Therefore, the demand for a better understanding of the underlying mechanisms of kidney damage and regeneration, for improving the current therapies as well as for the development of novel organoprotective and regenerative strategies in nephrology is bigger than ever.

The *Frontiers in Physiology* Research Topic collection “*Recent advances on renoprotection and kidney regeneration*” provides insight into several aspects of acute kidney damage and into the role of fibroblast growth factor 21 in chronic kidney disease. It also reports intriguing new findings on the function of the renin cells as a progenitor cell niche, on the therapeutic modulation of glomerular hemodynamics in the diabetic kidney, and on a kidney machine perfusion-based renoprotective drug discovery platform. Last but not least, an in-depth review focuses on the use of materials mimicking renal extracellular matrix in new kidney models.

Acute kidney injury (AKI) is a common disease that associates not only with high short-term mortality but also with long-term adverse outcomes including chronic kidney disease (CKD) and cardiovascular complications (Lameire et al., 2008; Mehta et al., 2015; Teo and Endre, 2017). AKI is also particularly interesting with regard to regeneration because it could be completely reversed by modern therapy (Legendre et al., 2013) thus underscoring the high regenerative capacity of the kidney. Yet renal functional and structural integrity could not always be restored after AKI, resulting in loss of kidney function and CKD. Therefore ongoing efforts focus on better understanding the molecular mechanisms of AKI and the development of novel regenerative and protective therapeutic approaches.

In this Research Topic collection, Buse et al. provide an overview of whole-cell sequencing findings in AKI. Particularly single-cell transcriptome sequencing represents a powerful new tool for studying in detail the global organ expression landscape. It is also a technique with immense diagnostic potential that heralds new therapeutic strategies for AKI and AKI to CKD transition. This technology will help to clarify the fundamental question regarding the pre-existence of an intratubular progenitor cell population such as the “scattered” tubular cells (Lindgren et al., 2011) being required for tubular cell repair or whether all surviving cells are able to acquire regenerative potential via dedifferentiation. Results from the studies being discussed in this mini-review suggest the latter mechanism. Single-cell sequencing experiments also provide additional insights into the sex-specific differences of AKI after ischemia-reperfusion, a phenomenon that has been consistently demonstrated clinically in many animal models.

The diagnosis of AKI relies currently on measuring serum creatinine and urine production. However, these functional parameters only indirectly reflect the underlying tissue damage (and regeneration) in AKI. Zou et al. tackle this issue by reviewing novel subclinical biomarkers of AKI. The authors address the problem of AKI as a hospitalization complication, which is usually associated with renal tubule injury. They focus on the issue of subclinical AKI, characterized by structural renal damage in the absence or minor changes in functional markers: serum creatinine, glomerular filtration rate, and urinary output. This review provides a critical assessment of a comprehensive list of markers of renal tissue damage. The development of renal tubule injury markers aims to remedy the lack of specificity, sensitivity, and timeliness of functional markers for the diagnosis of structural damage in AKI. The review summarizes the molecular nature, physiology, analytics, preclinical studies, and clinical performance as subclinical AKI markers for cystatin C, NGAL, KIM-1, IL-18, LFBAP, and TIMP2/IGFBP7. Two recent candidates, clusterin and PenKid are reviewed in more detail. Performance appears to be conditioned to the clinical setting and the major limitation for most biomarkers is the lack of specificity. Thus, the value of measuring AKI biomarkers still resides in their integration with clinical assessment.

Corridon addresses the therapeutic potential of transient overexpression of the mitochondrial enzyme isocitrate dehydrogenase 2 (IDH2) in a rodent AKI model using plasmid-based hydrodynamic gene delivery. IDH2 is the primary enzyme maintaining the mitochondrial antioxidant system, which is markedly altered in tubular epithelial cells after AKI and may be responsible for successful ischemic pre-conditioning. In these experiments, the author demonstrates successful renal gene delivery as well as upregulated expression of IDH2 after early hydrodynamic gene transfer. Being executed 1 h after AKI induction, IDH2 gene transfer ameliorated AKI-mediated increases in serum creatinine and urea nitrogen levels, while urine output increased as well as the mitochondrial membrane potential. Apparently, still many hurdles have to be overcome to provide a successful transition of these experimental results to the clinic.

Using an acute glomerulonephritis model Labes et al. study the role of inflammation because it is thought to be crucial for the transition from acute injury to CKD. This group studies the renoprotection conferred by Annexin A1, a glucocorticoid inducible protein. They employ an animal model of Annexin A1 deficiency to show its role as a mediator of the resolution phase of rapidly progressive crescent glomerulonephritis. Deficiency of Annexin A1 led to an aggravated course of the disease with a phenotype of severe non-resolving inflammation and accelerated fibrosis. Annexin A1-deficient mice exposed to the nephrotoxic serum model exhibited a larger number of damaged glomeruli, increased abundance of inflammatory cells (neutrophils, macrophages, and leukocytes), and alteration of renal lipid levels towards a pro-inflammatory profile. Transcriptome analysis revealed upregulation of pathways related to leukocyte activation and cytokine production and secretion in Annexin A1 deficient mice at day 10 after induction of nephritis. Finally, augmented expression of fibrotic markers was demonstrated by qPCR and tissue staining for collagen. These results are in good agreement with previous observations on the anti-inflammatory and anti-fibrotic roles of Annexin A1 in the kidney. The upregulation of Annexin A1 or its downstream effectors may represent a new target for renoprotective strategies.

Zhou et al. summarize the accumulating evidence for the role of Fibroblast Growth Factor 21 (FGF21) in CKD. FGF21 serum levels increase in CKD. Importantly these could be used as a predictor for declining kidney function. Particularly in diabetic nephropathy, FGF21 appears to be a very precise prognostic marker. FGF21 is produced by the liver and is upregulated in states of cellular energy deficit. In the diabetic kidney FGF21 appears to work protectively by improving lipid metabolism and alleviating oxidative stress, thus essentially limiting renal injury and fibrosis. It is still to be discovered why FGF21 increases in the non-diabetic forms of CKD. Another interesting aspect to be addressed by future studies is why if FGF21 works protectively in CKD it is not sufficient to halt disease progression. With this regard, there are already promising findings showing that therapy with FGF21 analogs exerts beneficial metabolic effects (for more detail see review references).

The juxtaglomerular renin-producing cells in the afferent arterioles (termed hereafter renin cells) have been known for a long time as the primary source of renin in circulation. Renin is the central regulatory factor within the plasma renin-angiotensin-system, which plays a fundamental role in the control of arterial blood pressure. However, in the last several years these cells were reproducibly found to have also progenitor and protective functions in the kidney (Pippin et al., 2013; Starke et al., 2015; Lachmann et al., 2017; Steglich et al., 2020). Arndt et al. expanded the knowledge on the progenitor role of the renin cells by demonstrating that there is a seningle-nephron feedback system, which drives the movement of the renin cells into the glomerulus where they change their phenotype to replace damaged glomerular cells. The authors used longitudinal intravital microscopy and 3D image reconstruction to quantify the injury-directed renin cell migration in laser-irradiated glomeruli of mouse kidneys. Interestingly the directed migration of renin cells starts almost 3 days after the initial glomerular damage. At the same time, the intraglomerular renin cell descendants remain in spatial contact with the juxtaglomerular renin cell niche. While providing extensive quantification of the migration process for the first time, the authors only speculate about the signal mediating the feedback between glomerulus and extraglomerular renin cells. Thus, the exact nature of the signal(s) driving the renin cell migration and differentiation after glomerular injury remains to be resolved.

The intravital imaging technique was used by the same group to study the therapeutic effects of renoprotective drugs on glomerular hemodynamics in a mouse model of diabetic kidney disease. Diabetes mellitus is the primary cause of irreversible loss of kidney function and renal replacement therapy (Tziomalos and Athyros, 2015; Alicic et al., 2017). Glomerular hyperfiltration is a major pathophysiological factor, particularly in the early phase of diabetic kidney disease. Counteracting the increase of glomerular filtration rate (GFR) is a standard renoprotective therapy in diabetic patients. Kroeger et al. studied the effects of angiotensin-converting enzyme (ACE) inhibitor enalapril or/and sodium glucose cotransporter 2 (SGLT2) inhibitor empagliflozin on single nephron GFR (snGFR) in diabetic mice using intravital imaging. Treatment with enalapril, empagliflozin, or a combination of both decreased snGFR. While the effect on snGFR was uniform, the different treatment modes induced distinct changes in the resistance of the corresponding afferent and efferent arterioles, which consistently resulted in decreased filtration. These findings demonstrated that the complexity of the pathophysiologic mechanisms causing glomerular hyperfiltration in diabetic patients offers also multiple options for beneficial modulation by renoprotective treatment regimens.

Allogeneic kidney transplantation is considered the most advanced present-day renal replacement therapy. A major limitation therein is the low number of donors available compared to the number of patients with ESRD. Therefore, transplant tissue care is decisive for increasing the number of transplantable kidneys. A modern approach in preserving transplant integrity is normothermic machine perfusion (NMP). Although still limitedly tested with human kidneys, NMP is expected to alleviate tissue ischemia and allow organ preconditioning. In this Research Topic collection, Hofmann et al. describe an *ex vivo* kidney slice perfusion as a further development of a rodent NMP technique previously reported by the same group (Czogalla et al., 2021). The novel perfusion method is based on a 3D-printed kidney slice incubator (KSI) with slots for up to nine kidney mouse slices. With this device, a high-throughput pipeline for drug testing was established. Since the testing conditions are very similar to the whole organ perfusion, coupling KSI to NMP would dramatically accelerate the preclinical studies on potential applications of NMP in human kidney allotransplantations. In support of this exciting perspective, the authors report the identification of β-nicotinamide-adenine-dinucleotide as an agent reducing endoplasmic reticulum stress in perfused kidneys through their rodent KSI-NMP pipeline.

Preclinical animal models fail to reproduce all the characteristics of AKI and CKD in humans. This is particularly evident and relevant in the field of nephrotoxicity. However, there is not a good alternative because traditional *in vitro* models based on the bi-dimensional culture of renal cells lack many of the biochemical and biomechanical stimuli present *in vivo*. Lacueva-Aparicio et al. summarize different strategies for improving *in vitro* models, based on the incorporation of biochemical and biomechanical properties of extracellular matrix (ECM) surrogates. Their thorough but concise review covers 2D, 2.5D, and 3D architecture models and discusses how biological and synthetic ECM influence renal phenotype and response to damage. Recent advances in the fields of organoids, decellularized ECM, bioprinting, and microfluidics are presented, to stress the need of incorporating complex architectures and biochemical ECM stimuli. The combination of these techniques will result in complex models close enough to *in vivo* kidney function and disease to allow for the substitution of animal preclinical models, facilitating the evaluation of nephroprotection, kidney damage, and regeneration.

In conclusion, the papers in this Research Topic collection not only summarize modern aspects of renoprotection but also report original findings on the regenerative capacity of the kidney and inspiring therapeutic approaches with strategic potential.
